# Basin Scale Variation on the Composition and Diversity of Archaea in the Pacific Ocean

**DOI:** 10.3389/fmicb.2017.02057

**Published:** 2017-10-23

**Authors:** Xiaomin Xia, Wang Guo, Hongbin Liu

**Affiliations:** Division of Life Science, The Hong Kong University of Science and Technology, Hong Kong, Hong Kong

**Keywords:** vertical profiles, marine archaea, oxygen minimum zone, methanogenic archaea, methanotrophic archaea

## Abstract

The Archaea are a widely distributed group of prokaryotes that inhabit and thrive in many different environments. In the sea, they play key roles in various global biogeochemical processes. Here, in order to investigate the vertical profiles of archaeal community across a large geographic distance, the compositions of archaeal communities in seven seawater columns in the Pacific Ocean were investigated using high throughput 454 pyrosequencing of the 16S rRNA gene. The surface archaeal communities showed lower diversity and greater variability than those in the deeper layers. Two of the major archaeal phyla that displayed different depth preferences were Thaumarchaeota and Euryarchaeota. The majority of Thaumarchaeota belonged to Marine Group I (MGI), which had high relative abundance in deep water. In contrast, Euryarchaeota, which mainly consisted of Marine Group II (MGII) and III (MGIII), were dominant in the surface layer. Compared with MGI and MGII, MGIII were less abundant in seawater and generally absent from the surface water of the subarctic Pacific. In addition, niche separation in the MGI, MGII, and MGIII subgroups was also observed. For example, MGI.C and MGII.A (the major subgroups of MGI and MGII, respectively) displayed a strong negative correlation with each other. The highest level of archaeal diversity was found in the core of an oxygen minimum zone (OMZ) located off Costa Rica, which resulted from the co-occurrence of both anaerobic and aerobic archaea. For example, methanotrophic archaea ANME-2, methanogenic archaea and several sediment origin archaea, such as Marine Benthic Group A (MBGA) and Bathyarchaeota, were all detected at relatively high abundance in the OMZ. Together, our findings indicate that vertical heterogeneities along water columns and latitudinal differentiation in the surface waters are ubiquitous features of archaeal communities in the Pacific Ocean, and the OMZ off Costa Rica is an archaeal biodiversity hot-spot.

## Introduction

The Archaea comprise one of the three domains of the living world, along with Bacteria and Eukarya (Woese and Fox, 1977; Woese et al., 1990). These single-celled microorganisms are widely distributed in the soil (Kudo et al., 1997), and in both fresh and coastal waters (DeLong, 1992; Schleper et al., 1997), as well as in some extreme environments, such as the polar regions (DeLong et al., 1994) and in hot springs (Barns et al., 1994; Takai and Horikoshi, 1999). In these various different environments, the Archaea play important roles in the global biogeochemical cycles, via a number of processes including carbon assimilation, mineralization, and precipitation (Berg et al., 2010; Offre et al., 2013). They also play a key role in various aspects of the nitrogen cycle (such as in nitrogen assimilation, Cabello et al., 2004; and ammonia oxidization, Könneke et al., 2005; Wuchter et al., 2006), as well as in sulfur cycling processes (such as sulfidogenesis and sulfide oxidation, Offre et al., 2013). Even though the diversity of the Archaea has been studied extensively in seawater (Molina et al., 2010; Alonso-Sáez et al., 2011; Sintes et al., 2013; Hugoni et al., 2015; Xia et al., 2015; Haro-Moreno et al., 2017), most of these studies only investigated archaeal community diversity in the upper water layers or in specific regional marine environments.

In the global ocean, it is estimated that the number of archaeal cells is approximately 1.3 × 10^28^ cells, which comprise >20% of the oceanic prokaryotes (Karner et al., 2001). Below the euphotic zone, this percentage increases to >50% (Karner et al., 2001). As most marine planktonic archaea cannot be maintained in culture, DNA-based molecular methods, such as terminal restriction fragment length polymorphism (TRFLP; Moeseneder et al., 2001) and clone library analysis (Massana et al., 2000; Bano et al., 2004), have been widely applied to study archaeal diversity in marine environments. These and other studies have revealed that in seawater, organisms from just two phyla make up the bulk of the archaeal communities. These phyla are Thaumarchaeota (formerly mesophilic Crenarchaea) and Euryarchaeota (Karner et al., 2001; Bano et al., 2004; Brochier-Armanet et al., 2008). Marine Group I (MGI), which are affiliated with the phylum Thaumarchaeota, consist of 35% of the total microbial cells in the deep ocean (Karner et al., 2001). At least some members of this group are involved in ammonia oxidization and are dominant ammonia-oxidizers in marine environments (Wuchter et al., 2006). It has also been suggested that MGI archaea are the main source of N_2_O in the ocean (Santoro et al., 2011). MGI are phylogenetically divided into four subgroups (I.A, I.B, I.C, and I.D), which display different distribution patterns and activities in the marine environment (Hugoni et al., 2013).

Marine Group II (MGII) are the dominant group of Euryarchaeota in seawater. Recent studies suggest that MGII Euryarchaeota might contribute significantly to the tetraether lipid pool in the ocean (Lincoln et al., 2014; Wang et al., 2015). In addition, they have been reported to be the dominant prokaryote in several phytoplankton blooms (Needham and Fuhrman, 2016); this supports the notion that MGII are closely linked to particulate organic matter (Orsi et al., 2015; Zhang et al., 2015). Two major subgroups of MGII (MGII.A and MGII.B) have been identified and these exhibit temporal and geographical variations of abundance in the marine surface water (Hugoni et al., 2013; Xia et al., 2015). Marine Group III (MGIII), defined by Fuhrman and Davis (1997), are also included in Euryarchaeota, but they have relatively low abundance in seawater. In addition to Euryarchaeota and Thaumarchaeota, other Archaea phyla, such as Korarchaeota and Nanoarchaeota, have been reported in seawater (Barns et al., 1994; Clingenpeel et al., 2013), but these are usually minor groups.

A recent study on the vertical profiles of bacteria in the tropical and subarctic oceans provided valuable insight into their distribution and diversity in marine water columns (Jing et al., 2013). However, a systematic investigation of the vertical and latitudinal variations of archaeal community composition in the global ocean has never been attempted. Bano et al. (2004) compared the composition of the archaeal communities in the Arctic and Antarctic Oceans, and found that in deep water there was significant overlap, but in the surface waters the communities were very different. They also reported that the composition of the archaeal community in the Arctic Ocean exhibited no seasonal variation (Bano et al., 2004). A subsequent study on the vertical distribution of the archaeal community composition clearly showed niche separation of Euryarchaetota and Thaumarchaeota, such that the former were more abundant in the surface waters, whereas the latter more dominant in the mesopelagic and bathypelagic deep waters (Herndl et al., 2005). In some specific marine environments, such as the oxygen minimum zone (OMZ) (Belmar et al., 2011; Ulloa et al., 2013) and the Black Sea (Schubert et al., 2006), unique archaeal communities have been reported. However, until now, most of the studies on marine archaeal diversity have been conducted using low resolution methods, such as clone library analysis. Only limited studies using the higher resolution high through-put pyrosequencing technique were conducted for the archaea communities in the northwestern Pacific Ocean (Brown et al., 2009) and in the surface water of the South China Sea (Xia et al., 2015). To investigate the vertical and horizontal distributions of archaea in the Pacific Ocean, we collected water samples from seven geographically-separated oceanic stations and applied 454 pyrosequencing targeting the 16S ribosomal RNA (rRNA) gene. The aims of this study were: (1) to reveal the vertical profiles of the archaeal communities across a large geographic distance to help us understand the spatial distribution and heterogeneity of archaea in the global ocean; (2) to test our hypothesis that on a global scale, the microbial community structures are more variable in the surface waters than they are in the deeper layers due to bigger changes in the physico-chemical environment occurring in the former; and (3) to investigate the archaeal diversity in the OMZ.

## Materials and methods

### The study region and collection of seawater samples

**Figure 1:**
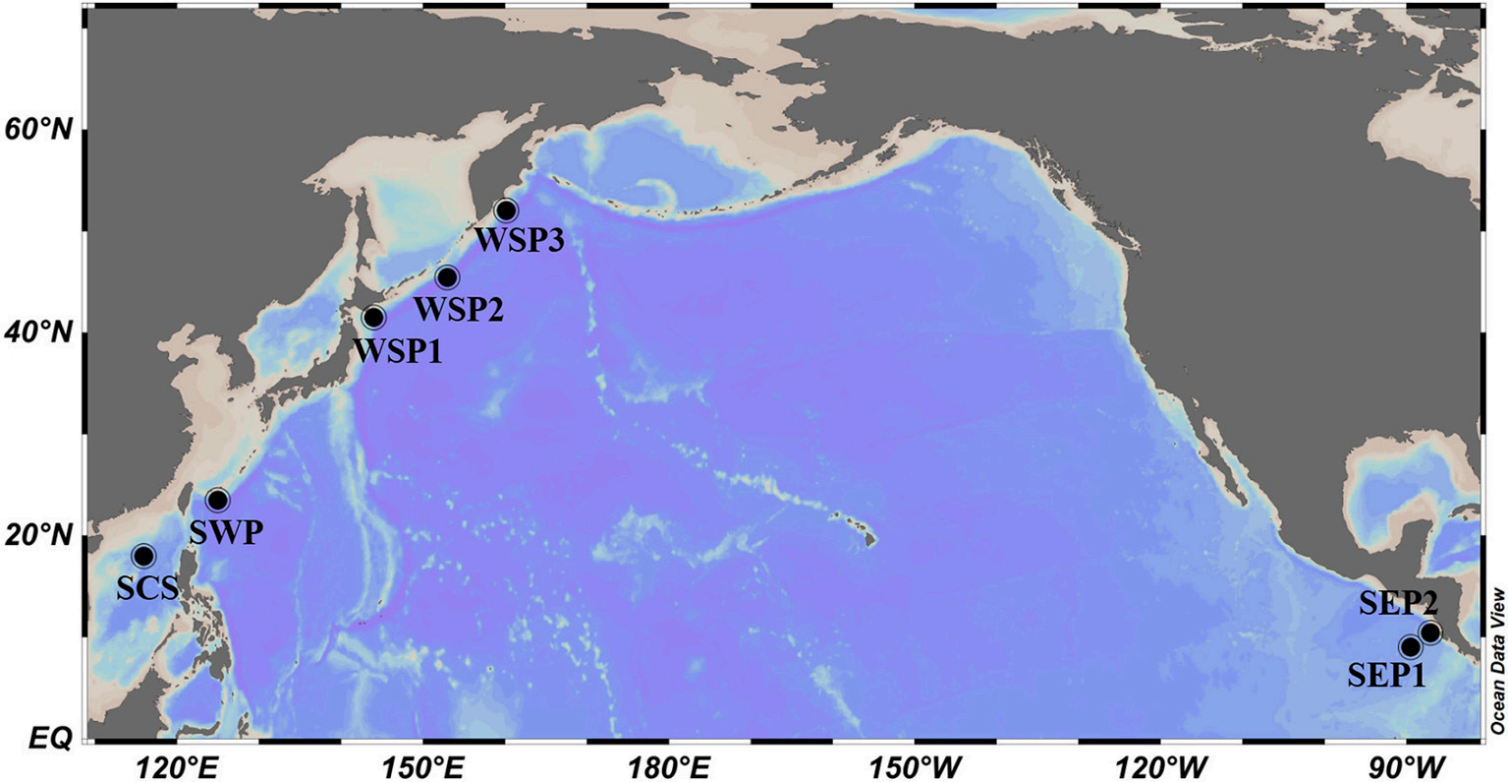
Sketch map showing the various sampling locations. SCS, South China Sea; SWP, Subtropical Western Pacific-Station; SEP, Subtropical Eastern Pacific; WSP, Western Subarctic Pacific.

A total of 26 seawater samples were collected from different layers at seven stations in the Pacific Ocean (Table 1, Figure 1). Stations SCS and SWP are located in the subtropical western Pacific Ocean, stations SEP1 and SEP2 are in the subtropical eastern Pacific Ocean, and stations WSP1, WSP2, and WSP3 are in the western subarctic Pacific Ocean (Figure 1). Water samples (approximately 4 L) were collected using a CTD carousel water sampler with clean plastic Niskin sampling bottles, pre-filtered through 3 μm pore size polycarbonate (PC) membranes (47 mm diameter, Millipore), and then filtered onto 0.22 μm PC membranes (47 mm diameter, Millipore). All the samples were collected during the months of May, June and July, and kept at −80°C until required for use.

**Table 1:** Characteristics of the sampling stations.

**Station name**	**Sampling time**	**Location**		**Sampling depth (m)**	**Temperature**	**Salinity (ppt)**	**Dissoved oxygen (μmol/L)**
		**Longitude (E)**	**Latitude (N)**				
South China Sea (SCS)	July, 2009	115.96	17.99	5	28.62	33.25	202.60
				100	19.42	34.45	118.00
				500	8.99	34.42	97.10
				2,000	2.49	34.6	105.20
Subtropical Western Pacific (SWP)	July, 2013	125	23.5	10	28.32	34.56	219.78
				200	18.50	34.77	NA
				1,500	2.47	34.56	NA
Subtropical Eastern Pacific-Station 1 (SEP1)	July, 2010	−90.504	9.04	40	15.38	34.82	19.22
				200	11.90	34.80	15.87
				600	6.82	34.59	1.00
				2,000	2.28	34.65	88.45
Subtropical Eastern Pacific-Station 2 (SEP2)	July, 2010	−92.917	10.416	5	27.94	33.70	191.98
				500	8.34	34.62	0.74
				1,000	4.48	34.57	15.92
Western Subarctic Pacific-Station 1 (WSP1)	May, 2011	144	41.5	10	4.94	33.12	NA
				100	1.85	33.17	NA
				500	3.33	34.08	NA
				1,500	2.35	34.50	NA
Western Subarctic Pacific-Station 2 (WSP2)	June, 2014	152.967	45.433	5	7.31	32.83	342.90
				200	2.89	33.56	139.70
				500	3.23	34.06	34.50
				3,000	1.63	34.69	120.40
Western Subarctic Pacific-Station 3 (WSP3)	June, 2014	160.167	52.01	5	5.95	32.78	344.00
				150	2.00	33.30	233.10
				500	3.49	34.17	14.20
				3,000	1.56	34.67	125.20

*NA, not available*.

### DNA extraction, PCR amplification and high-throughput pyrosequencing

The total genomic DNA of the filtered water samples was extracted using a PureLink® Genomic DNA Mini Kit (Invitrogen, CA), according to the manufacturer's instructions. Total DNA was eluted in 50 μL elution buffer and stored at −80°C until required.

Barcoded universal primers 340F (5′-adaptor + barcode + CCCTAYGGGGYGCASCAG-3′) and 1000R (5′-adaptor + GGCCATGCACYWCYTCTC-3′) (Gantner et al., 2011) were used for archaeal 16S rRNA gene amplification. One aliquot of 25 μL PCR reaction mixture included: 1 μL forward and reverse primers (10 μM), 2.5 μL 10 × PCR buffer (Invitrogen, CA), 0.75 μL MgCl_2_ (50 mM), 0.5 μL dNTP mix (10 mM, Invitrogen, CA), 0.2 μL Platinum Taq DNA Polymerase (Invitrogen, CA), 1 μL genomic DNA and 18.3 μL sterilized ddH_2_O. The PCR reactions were performed in triplicate under the following conditions: an initial denaturation step at 95°C for 5 min; then 35 cycles at 95°C for 45 s, 55°C for 1 min, and 72°C for 1 min; and a final elongation step at 72°C for 7 min. A negative control was also performed during amplification to assess for possible contamination. The PCR products of each sample were combined and then purified via an Invitrogen PureLink® Quick Gel Extraction Kit (Invitrogen, CA). The purified PCR products were quantified using a Quant-iT™ PicoGreen assay (Invitrogen, CA), and then mixed in equal amounts with each other and sequenced in a two-region 454 run on a GS PicoTiterPlate using a GS Junior pyrosequencing system, according to manufacturer's instructions (Roche, 454 Life Sciences, Branford, CT, USA).

### Data analysis

In total, 381,263 raw sequences were obtained by 454 pyrosequencing. The sequences were separated based on their forward primer with identifiers (MIDs). All retaining reads completely matched the barcodes, with a maximum of a single mismatch to the primers. The sequencing adaptor and barcodes were then removed, and the sequences were de-noised with the command *shhh.seqs* (sigma = 0.01) using Mothur (Schloss et al., 2009), after which short sequences (i.e., <300 bp) and low quality sequences (i.e., those with an average quality score <25, or containing ambiguous bases, or containing maximum homopolymers of 8 bp) were trimmed. Chimeric sequences (comprising 5.5% of total sequences) identified using command *chimera.uchime* were also removed. A taxonomic identification of each read was conducted using Mothur against the Greengene database (http://greengenes.lbl.gov/cgi-bin/nph-index.cgi) at a cutoff value of 80. Sequences that were categorized as being unclassified archaea (i.e., exhibiting <80% similarity with sequences in the Greengene database) were also removed. The remaining sequences were clustered into operational taxonomic units (OTUs) at a 0.03 dissimilarity level. The OTUs containing only one sequence were removed. Subsequently, 1,691 sequences were subsampled from each sample for further analysis. The sequencing coverage and number of OTUs were summarized using Mothur. The species richness, evenness, and Shannon diversity were calculated at 97% sequence similarity using Primer 5 (Primer-E-Ltd, UK). Nonmetric Multidimensional Scaling (NMDS) analysis was applied to compare the archaeal community composition among all the samples. The similarity between archaeal communities was also calculated using Primer 5. The representative sequences of the 100 most abundant OTUs were randomly extracted using Mothur and then used to construct a Maximum Likelihood phylogenetic tree. The tree was constructed using MEGA 6 (Tamura et al., 2013) based on the Kimura 2-parameter model selected by Modeltest. The relative abundance of the 100 most abundant OTUs was square-root transformed and used to generate a heatmap by Heml (Deng et al., 2014). The Spearman correlation between archaeal groups was calculated using R package Cor (Team, 2013). Only strong correlations (*r* > 0.6 or < −0.6, and *P* < 0.01) were considered and visualized through net-work analysis using Cytoscape (Shannon et al., 2003).

### Sequence submission

All the sequences obtained from this study have been deposited in the National Center for Biotechnology Information (NCBI) Sequence Read Archive (SRA) (Table 2).

**Table 2:** Diversity estimates for water samples collected from seven sites in the North Pacific Ocean obtained by 454 pyrosequencing.

	**No. of sequences**	**No. of OTUs**	**Species richness**	**Evenness**	**Shannon diversity**	**Coverage**	**Accession number**
SCS-5m	1691	15	1.883	0.7174	1.943	1	SRR5349124
SCS-100m^*^	1691	83	11.030	0.4985	2.203	0.97	SRR5349123
SCS-500m	1691	77	10.220	0.5074	2.204	0.98	SRR5349122
SCS-2000m	1691	75	9.956	0.3685	1.591	0.97	SRR5349121
SWP-10m	1691	21	2.691	0.6456	1.966	1	SRR5349112
SWP-200m	1691	75	9.956	0.5825	2.515	0.99	SRR5349111
SWP-1500m	1691	83	11.030	0.4094	1.809	0.97	SRR5349110
SEP1-40m	1691	27	3.498	0.6058	1.997	1	SRR5349113
SEP1-200m	1691	77	10.220	0.5751	2.498	0.98	SRR5349114
SEP1-600m	1691	22	2.825	0.6083	1.880	0.98	SRR5349115
SEP1-2000m	1691	66	8.745	0.4087	1.712	1	SRR5349116
SEP2-5m	1691	20	2.556	0.6622	1.984	1	SRR5349105
SEP2-500m	1691	68	9.014	0.6979	2.945	0.99	SRR5349103
SEP2-1000m	1691	31	4.036	0.5169	1.775	0.99	SRR5349104
WSP1-10m	1691	15	1.883	0.3601	0.975	1	SRR5349120
WSP 1-100m	1691	22	2.825	0.3826	1.183	0.99	SRR5349119
WSP 1-500m	1691	47	6.189	0.5832	2.246	0.99	SRR5349118
WSP1-1500m	1691	47	6.189	0.6161	2.372	0.99	SRR5349117
WSP2-5m	1691	16	2.018	0.4092	1.134	1	SRR5349109
WSP2-200m	1691	67	8.879	0.5398	2.27	0.98	SRR5358886
WSP2-500m	1691	58	7.668	0.6301	2.559	0.99	SRR5349108
WSP2-3000m	1691	56	7.399	0.4634	1.866	0.99	SRR5349102, SRR5349101
WSP3-5m	1691	13	1.614	0.2776	0.7121	1	SRR5349107
WSP3-150m	1691	45	5.919	0.493	1.877	0.99	SRR5349098, SRR5349097
WSP3-500m	1691	44	5.785	0.6263	2.37	0.99	SRR5349106
WSP3-3000m	1691	60	7.937	0.4396	1.8	0.98	SRR5349100, SRR5349099

*SCS, South China Sea; SWP, Subtropical Western Pacific-Station; SEP, Subtropical Eastern Pacific; WSP, Western Subarctic Pacific*.

* *The raw sequence number of this sample is 94,257 which is far more than other samples, thus we subsampled 10,000 reads from this sample for subsequent analysis*.

## Results

### Alpha diversity

In total, 505 OTUs at a 97% sequence similarity threshold were obtained from the 26 samples. The number of OTUs ranged from 13 to 83 across all the samples, with the surface waters harboring significantly lower numbers of OTUs than the other layers. The species richness was also relatively lower in the surface samples (Table 2). Along the water columns, the Shannon diversity and evenness indices were the highest in the intermediate water layers (i.e., at 100–500 m). The archaeal species richness did not show clear differences among the samples collected in the same layers at different locations. However, archaeal diversity (Shannon index) was higher in the subtropical warm water than in the subarctic Pacific (Table 2). The highest Shannon diversity index (of 2.945) occurred at SEP2-500 m, which is the core of the OMZ off Costa Rica in the eastern subtropical Pacific Ocean. The sequencing coverage value for all the samples was >0.97.

### Similarity between the archaeal communities

To compare the composition of the archaeal communities in the different depths of water, samples were arranged into four groups according to their sampling depth [i.e., surface (0–10 m); subsurface (40–200 m); mesopelagic (500–1,000 m); and bathypelagic (1,000–3,000 m)]. The similarity among samples within the surface and subsurface groups was 31.54 and 23.98%, respectively; this is approximately half of that in the mesopelagic (56.36%) and bathypelagic (67.12%) groups (Table 3). The highest Shannon diversity for the archaeal community occurred in the subsurface group (Shannon index = 3.00), whereas the lowest was in the bathypelagic group (Shannon index = 2.10) (Table 3). Although individual samples of the surface group had a lower archaeal diversity than the other samples (Table 2), the diversity of the surface group was not the lowest (Table 3). A SIMPER analysis was performed to calculate the dissimilarities among the groups of the different layers (Table 4), and this revealed that the archaeal community structure of the surface group was significantly different from that in the other layers, with dissimilarities >90.00%. The highest dissimilarity value (dissimilarity = 98.48%) was obtained when comparing the surface and bathypelagic groups, whereas the lowest value was obtained when comparing the mesopelagic and bathypelagic groups (dissimilarity = 43.30%).

**Table 3:** Similarity and Shannon index in each layer group estimated based on the 16S rRNA gene.

**Layers**	**Similarity**	**Shannon Index**
Surface	31.54	2.21
Subsurface	23.98	3.00
Mesopelagic	56.36	2.74
Bathypelagic	67.12	2.10

*Surface (0–10 m); subsurface (10–200 m); mesopelagic (200–1,000 m); and bathypelagic (1,000 m-bottom)*.

**Table 4:** Dissimilarity index among the different layer groups estimated based on the 16S rRNA gene.

**Dissimilarity**	**Surface**	**Subsurface**	**Mesopelagic**
Subsurface	94.08		
Mesopelagic	97.75	69.41	
Bathypelagic	98.48	73.95	43.30

**Figure 2:**
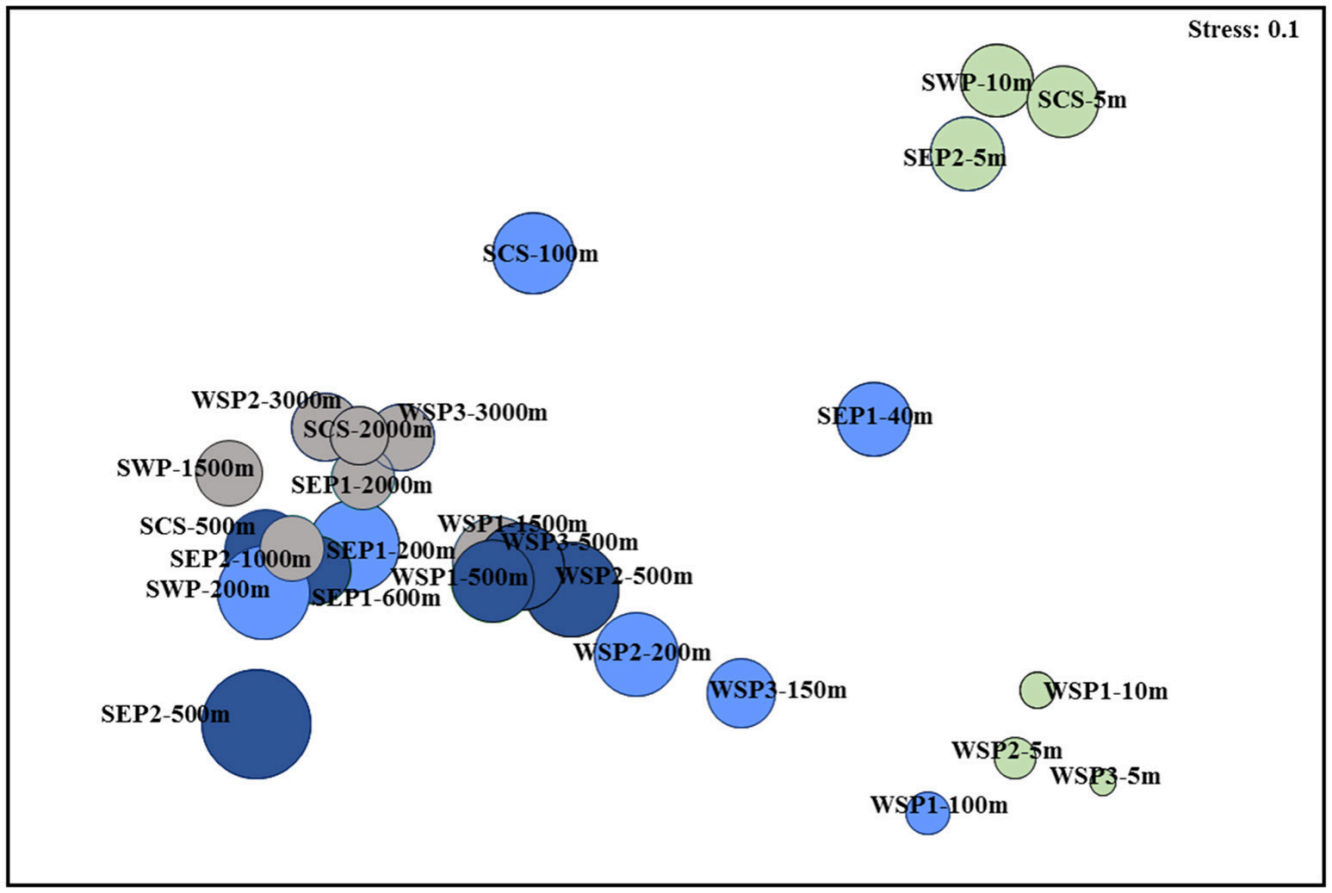
Relationship among the archaeal communities in the water samples collected from the seven sites in the Pacific Ocean demonstrated by the NMDS plot. The surface samples are indicated in light green; the subsurface samples are in light blue; the mesopelagic samples are in dark blue; and the bathypelagic samples are in gray.

The NMDS analysis showed that samples from the mesopelagic and bathypelagic layers clustered together even when they were from different oceanic regions, but they were distinct from samples from the surface and subsurface layers (Figure 2). The surface archaeal communities formed two distinct groups, one group was composed of samples from the subarctic waters, and the other group was made up of samples from the subtropical warm waters. The NMDS analysis results also supported those from the SIMPER analysis, which indicated that archaeal communities in the surface and subsurface layers exhibited greater dissimilarity than they did in the mesopelagic and bathypelagic layers.

### Archaeal community structure

**Figure 3:**
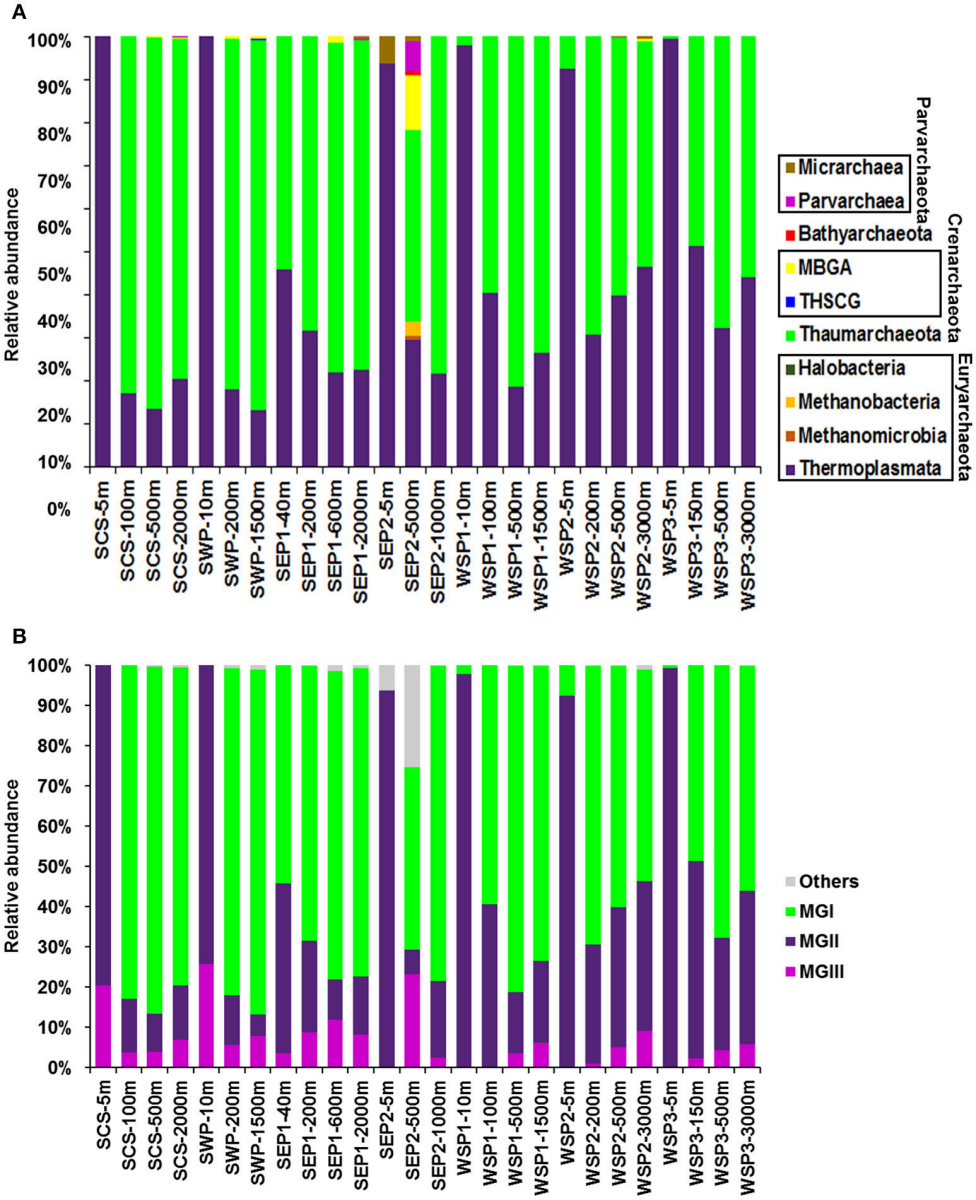
The relative abundance of the archaeal taxonomic groups in each sample. **(A)** Sequences were identified at the phylum level based on the Greengene database. **(B)** The relative abundance of each phylogenetic group.

Five archaeal phyla, Bathyarchaeota [previously named the Miscellaneous Crenarchaeotal Group (MCG)], Crenarchaeota, Euryarchaeota, Parvarchaeota, and Thaumarchaeota were identified in this study (Figure 3A). The surface archaeal communities were dominated by Euryarchaeota, whereas in the deeper layers Thaumarchaeota were predominant. Indeed, in samples from SCS-5 m and SWP-10 m, which were collected from the surface waters of the western Pacific Ocean, the relative abundance of Euryarchaeota even reached 100%. In contrast, Bathyarchaeota, Crenarchaeota, and Parvarchaeota, were not abundant in seawater. Bathyarchaeota were only found in SEP2-500 m (1.01% of the sample reads), which was in the core of the OMZ off Costa Rica. Crenarchaeota and Parvarchaeota were also mainly found in the SEP2-500 m sample.

At the class level, Thermoplasmata, Methanobacteria, Methanomicrobia, Halobacteria, MBGA (Marine Benthic Group A), THSCG (Terrestrial Hot Spring Crenarchaeotic Group), Thaumarchaeota MGI, Parvarchaea and Micrarchaea were found (Figure 3). Thermoplasmata and Thaumarchaeota MGI were the major archaeal classes, with the former being more abundant in the surface water, and the latter being dominant in the deep water. Methanobacteria and Methanomicrobia only occurred in the oxygen-depleted water of SEP2-500 m, with relative abundances of 3.52 and 0.89%, respectively. The MBGA were detected in nine samples but displayed a relatively high abundance in SEP2-500 m (12.58%). The THSCG were only found in SWP-200 m and SWP-1500 m. In addition, Halobacteria (another class of the phylum Crenarchaeota) only contained one sequence in this study and were found in SEP1-200 m. Moreover, Micrarchaea and Parvarchaea, which are the classes of the phylum Parvarchaeota, mainly occurred in the surface and 500 m depth waters, respectively, of station SEP2.

Three well-recognized marine archaeal groups, MGI, MGII, and MGIII, were retrieved from our samples (Figure 3B). MGI and MGII displayed a large heterogeneity with depth. MGI were more abundant in the deep water samples, with relative abundances ranging between 44.18 and 86.22%. In the surface waters, however, this group only accounted for 0–7.57% of the sample reads, and it was not detected at all in SCS-5 m and SWP-10 m. In contrast, MGII dominated in the surface samples (with 74.22–99.41% of the sample reads) and were more abundant in the subtropical waters of the Pacific than in the subarctic region. MGIII were less abundant than MGI and MGII, and in the subarctic surface water this group was rarely detected (Figure 3B).

**Figure 4:**
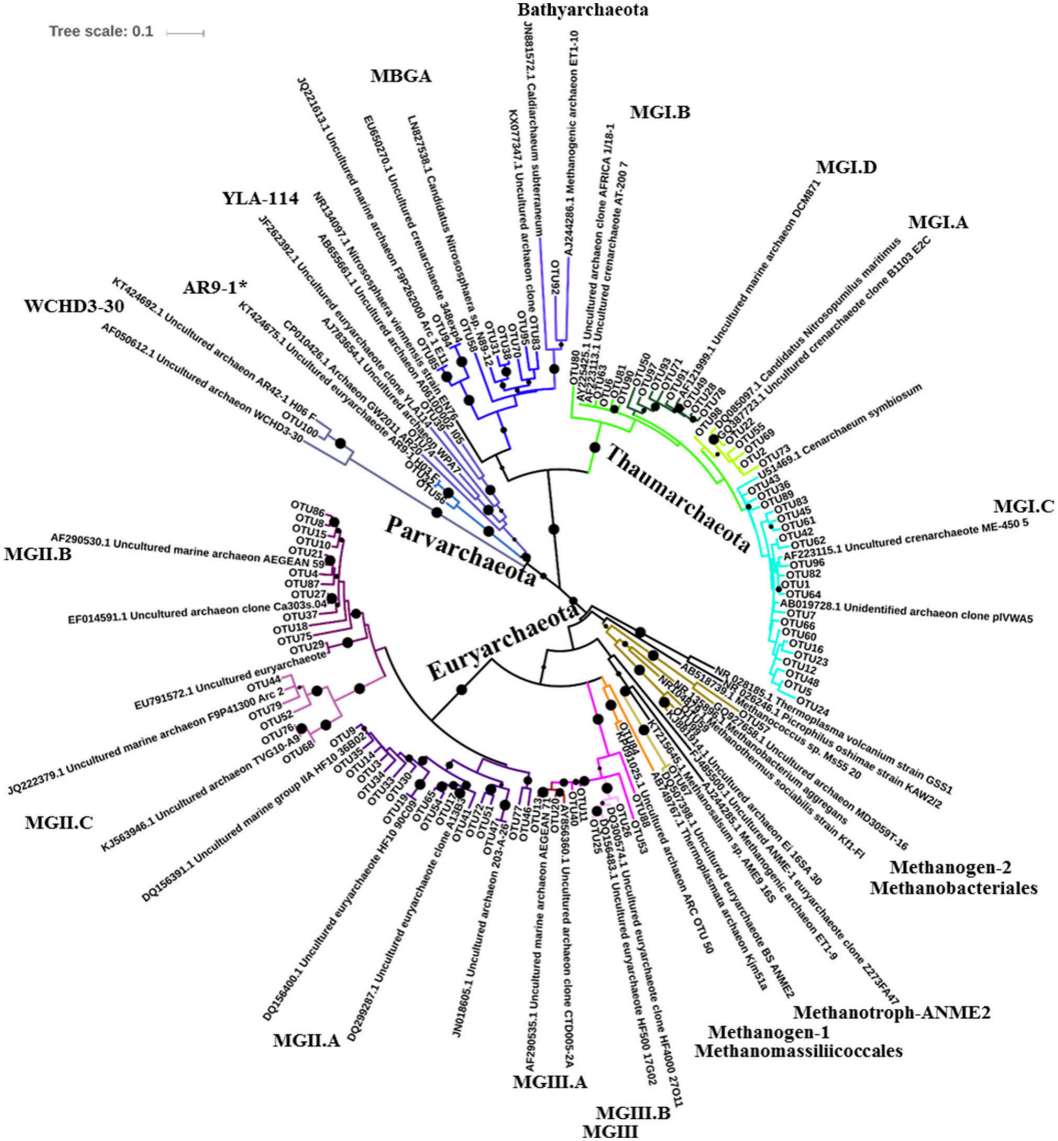
Maximum likelihood phylogenetic tree of the archaeal 16S rRNA gene sequences (representative sequences of the top 100 abundant OTUs). Dots over the nodes represent branches with bootstrap values >50%. The different archaeal groups are indicated by the different colors.

Phylogenetic analysis of the archaeal 16S rRNA gene sequences showed that Parvarchaeota, Euryarchaeota, and Thaumarchaeota formed three separate clusters (Figure 4). Most of the OTUs were affiliated with Thaumarchaeota MGI (40 OTUs) and Euryarchaeota MGII (34 OTUs). Four subgroups of MGI (i.e., I.A, I.B, I.C, and I.D; defined by Hugoni et al., 2013) were found in our study. Two major subgroups of MGII (i.e., MGII.A and II.B) were also detected. In addition to these two subgroups, some of the MGII sequences formed an independent clade (named MGII.C; Figure 4). We found that MGII.A contained more OTUs than the other two subgroups. MGIII, which also belong to the phylum Euryarchaeota, were composed of MGIII.A, MGIII.B and MGIII others. The diversity of MGIII was markedly lower than MGII. Two groups of methanogens as well as one group of methanotrophs were also included in Euryarchaeota. Methanogen-I consisted of sequences affiliated with Methanomassiliicoccales, and methanogen-II were composed of sequences from Methanobacteriales. For the two methanotrophic groups, ANME1 and ANME2 (Michaelis et al., 2002), only the ANME2 group was detected. Members of the class Parvarchaea (consisting of YLA114, WCHD3-30, and AR9-1; the latter being a new group named in this study) were also not abundant in seawater. In the phylogenetic tree, MBGA (containing 5 OTUs) and Bathyarchaeota (containing 1 OTU) were placed in the same cluster, but separated from the cluster formed by the MGI sequences, which were also minor groups in seawater.

**Figure 5:**
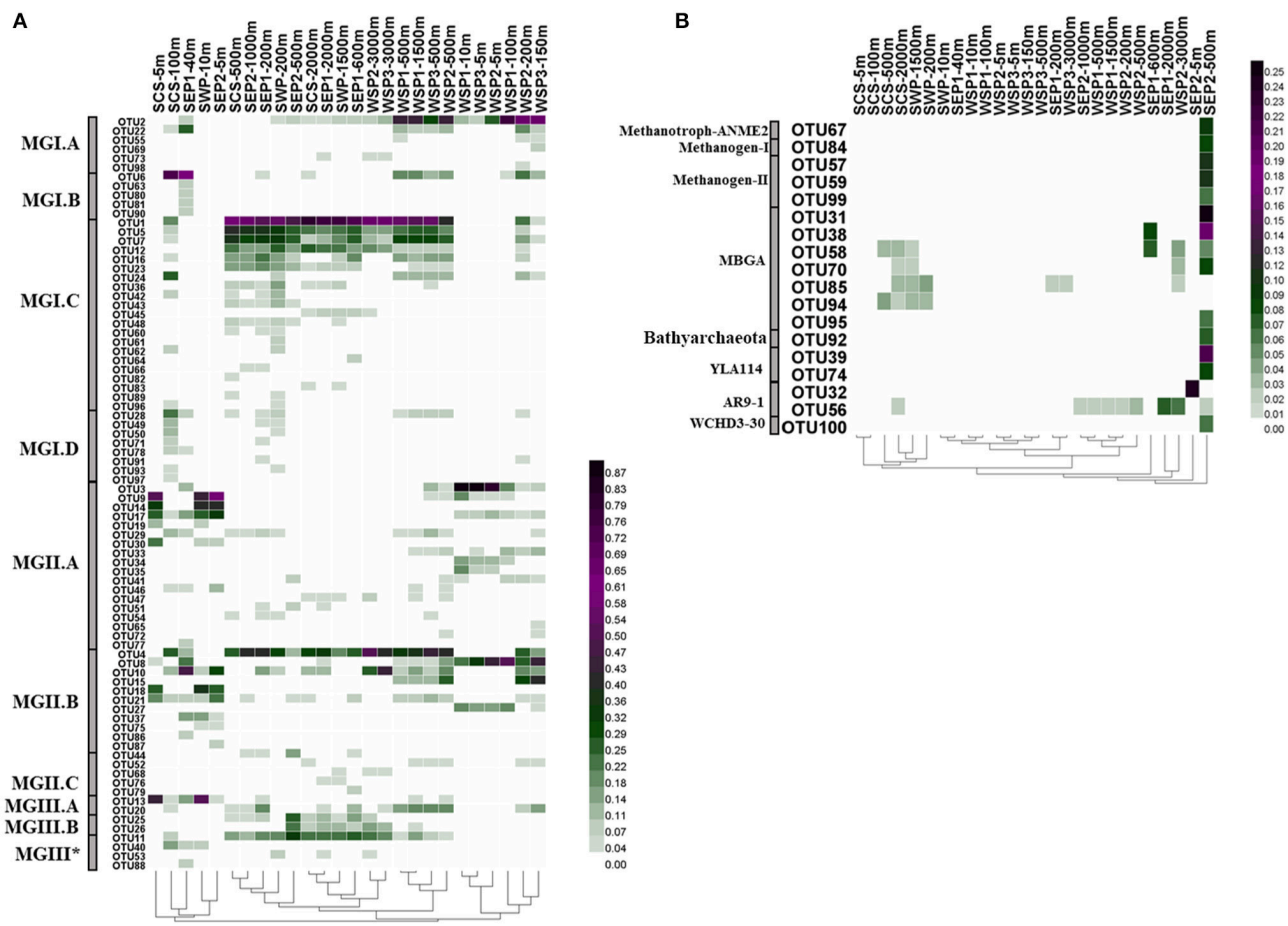
Heatmaps showing the distribution of the top 100 abundant OTUs in the various samples. **(A)** OTUs are classified as MGI, MGII, and MGIII archaea. **(B)** OTUs are affiliated with Methanotroph-ANME2, Methanogen-I, Methanogen-II, MBGA, Bathyarchaeota, YLA114, AR9-1, and WCHD3-30. The color bar indicates the relative abundance of each OTU (square root transformed). The classification of each OTU was based on the data shown in Figure 4. MGIII^*^ indicates OTUs not included in MGIII.A and MGIII.B.

Hierarchical cluster analysis of the 100 most abundant OTUs revealed biogeographic and niche-specific clustering patterns of the archaeal lineages (Figure 5). In total, 40 OTUs, including the two most abundant OTUs (OTU1 and OTU2), were identified as MGI archaea. Subgroups of MGI displayed different geographic distributions. MGI.C were more diverse and more abundant than MGI.A or MGI.B. They were widely distributed in the deep layers, and displayed no latitudinal variation in its relative abundance. In contrast, MGI.A and MGI.B were mainly distributed in the subsurface and mesopelagic layers. In addition, MGI.A were more abundant in the subarctic ocean, whereas MGI.B had a higher relative abundance in the low latitude subtropical regions. In the surface water, MGI archaea were found to be mainly contributed by MGI.A. MGI.D had the lowest abundance and the narrowest distribution in seawater, and were mainly detected in SCS-100 m. Compared with MGI, the MGII archaea had a relatively wider distribution and were found to be abundant in both the surface and deep waters. One of the major subgroups of MGII, MGII.A, was mainly distributed in the surface water but the major OTUs of MGII.A (i.e., OTUs-3, -9, -14, and -17) exhibited different geographic distributions. For example, OTU3 had a higher relative sequence abundance in the subarctic cold water, whereas the other OTUs were dominant in the subtropical warm water. Another major MGII subgroup, MGII.B, was dominant in the deeper layers, and OTU4 (the most abundant OTU of MGII.B), could be detected in the deep water of all the stations. Other MGII.B OTUs, OTU10 and OTU15, were also widely distributed in the deep layers, but they appeared to prefer the subarctic cold water. In contrast, some MGII.B archaea, such as OTUs-8, -18, -21, and -27, were found mainly in the surface water. In comparison to MGII.A and MGII.B, MGII.C were not abundant in seawater at any depth or location and present mainly in the mesopelagic and bathypelagic layers. In addition, MGIII exhibited a lower diversity and abundance than MGI and MGII. They were only distributed in low latitude regions; for example, the relative abundance of MGIII in SWP-10 m and SCS-5 m was 25.78 and 20.40%, respectively. A separation of the MGIII OTUs by different depths was also observed.

Methanogen-I, -II, Methanotroph-ANME2, MBGA, Bathyarchaeota, WCHD3-30, AR9-1, and YLA-114 were not abundant in any of the samples, apart from in SEP2-500 m, which was collected from oxygen-depleted water (Figure 5B).

**Figure 6:**
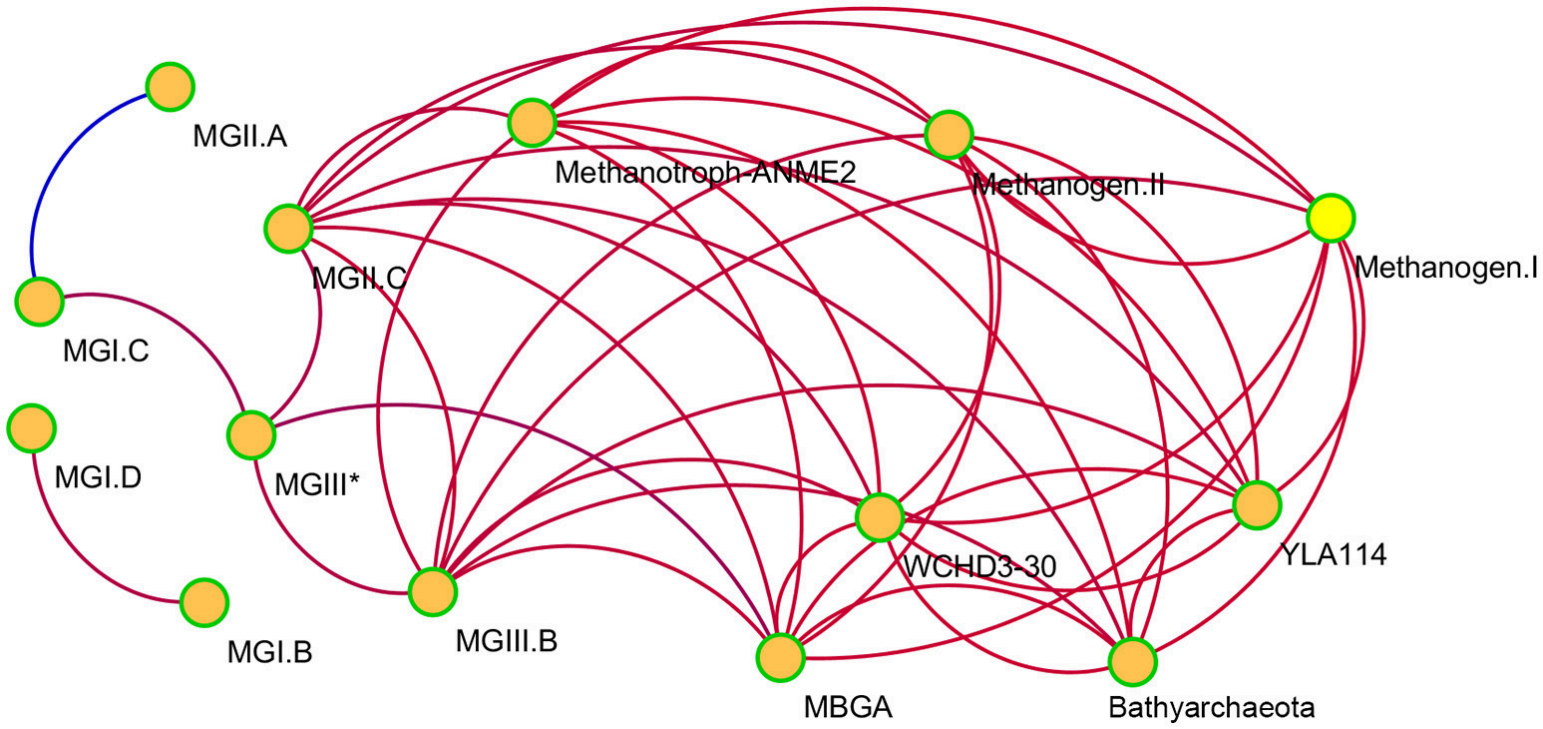
Co-occurrence network of archaeal groups based on correlation analysis. A connection stands for a strong (Spearman's *r* > 0.6 or < −0.6) and significant (*P*-value < 0.01) correlation. The red and blue lines indicate positive and negative correlation, respectively.

The 18 archaeal groups identified by phylogenetic analysis (Figures 4, 5) were selected to generate correlation patterns. We identified a network of 14 archaeal groups with 41 positive correlations and 1 negative correlation (Figure 6). MGII.C, MGIII.B, Methanotroph-ANME2, Methanogen-I and -II, MBGA, Bathyarchaeota, WCHD3-30, and YLA114 were linked by positive interactions. In addition, MGI.B and MGI.D positively correlated with each other, but did not link to other archaeal groups. The only negative correlation occurred between MGII.A and MGI.C.

## Discussion

### Low diversity of surface archaeal community

The occurrence of archaea in seawater was first reported by DeLong (1992) and Fuhrman et al. (1992). Subsequent studies revealed that archaea are ubiquitously distributed in marine environments and are involved in various biogeochemical processes. Archaea are known to have a low abundance in the surface waters of the open ocean (Karner et al., 2001), and have a relatively higher abundance in the deep ocean. Here, our results also revealed that the diversity and species richness of the archaeal community were lowest in the surface water and highest in the subsurface and mesopelagic layers (Table 2). This is similar to the distribution pattern of bacteria, which has been shown to have a higher richness and diversity in the intermediate layer (Jing et al., 2013). These results suggest that such a vertical distribution pattern might be a general feature of prokaryotes in the global oceans. The low archaeal diversity observed in the surface water might be due to the fact that the growth of some archaea (such as ammonium-oxidizing archaea) can be directly inhibited by light (Erguder et al., 2009). In a previous study, it was suggested that planktonic marine bacteria exhibit a latitudinal gradient of increasing diversity (species richness) from the poles toward the equator (Fuhrman et al., 2008). However, in terms of species richness, our data showed that archaea in the different latitudinal regions were somewhat similar (Table 2). This might suggest that the general geographic richness pattern described in previous reports, does not match the distribution of archaea in the global ocean. It is also worth noting that the highest diversity in the archaeal community occurred at a depth of 500 m in the Costa Rica Dome, an oxygen-depleted environment (Table 2). This is consistent with previous studies, which demonstrated that both the ammonia oxidation archaea (AOA, Molina et al., 2010) and total archaeal communities (Belmar et al., 2011) have a higher diversity in oxygen-deficient zones, when compared with the surface oxic zone. It has been suggested that the co-occurrence of anaerobic and aerobic processes in oxygen-depleted zones might explain this high level of archaeal diversity (Füssel, 2014).

### Heterogeneity of surface archaeal communities

Studies on marine bacteria have revealed that the composition of bacterial communities in the surface water is relatively less stable than in the deep water (Jing et al., 2013). This is thought to be due to fluctuations in environmental factors, such as wind and sunlight, which only affect the surface water (Bryant et al., 2016). With regards to the composition of the archaeal community, our new data also clearly showed that there was more variation in surface water than in deep waters (Table 2). These therefore add to the accumulating evidence highlighting the heterogeneity of the microbial community as a whole in the surface waters of the marine environment (Jing et al., 2013). Our observations were also supported by the NMDS analysis (Figure 2). We also showed that the surface archaeal communities in the subarctic water formed a cluster that was separate from the cluster formed by the subtropical water communities, which suggests a possible latitudinal differentiation of the surface archaeal community composition. This finding is consistent with previous studies that reported different archaeal communities in the North (Brown et al., 2009) and South Pacific Gyres (Yin et al., 2013). However, we noted that samples from the western Pacific Ocean (SWP-10 m and SCS-5 m) were closely grouped with the sample from the eastern Pacific Ocean (SEP2-5 m), which suggests that the archaeal community might have little longitudinal variation in community composition.

### Differentiation in vertical distribution of dominant archaeal groups

Here, we observed a vertical differentiation of the archaeal community in water columns. Our observations are consistent with those from studies in the Santa Barbara channel (Massana et al., 1997) and eastern Mediterranean Sea (De Corte et al., 2009), where depth-related differences in the archaeal community composition were also reported. In the former, Euryarchaea were reported to dominate in the surface waters, whereas Thaumarchaea (formerly mesophilic Crenarchaea) were more abundant in the deep layers (Massana et al., 1997). Similarly, we showed that all the surface samples were dominated by Euryarchaeota, with a relative abundance > 90% (Figure 3). We also showed that Euryarchaeota in the marine surface water, were mainly contributed by MGII. The potential importance of MGII archaea in carbon cycling in the global ocean was described by Lincoln et al. (2014) and Zhang et al. (2015), and these are also recognized as being significant contributors of tetraether lipids to the ocean. The MGII archaea were previously divided into two distinct phylogenetic lineages, II.A and II.B (Hugoni et al., 2013), which show seasonal variations in abundance in marine surface waters. For example, in the SCS, MGII.A are the dominant lineage in the summer, whereas MGII.B are more abundant in the winter (Table 5; Xia et al., 2015). A similar pattern was also reported in the NW Mediterranean Sea (Table 5; Galand et al., 2010). Similarly, our results showed that in the summer, MGII.A were the dominant archaea in the marine surface water of both the cold subarctic ocean and the warm tropical/subtropical ocean. This suggests that the seasonal variation in abundance of MGII.A and MGII.B might be a global distribution pattern. Moreover, our results also clearly demonstrated different distributions of MGII.A and MGII.B along the vertical water columns. MGII.A were mainly restricted to the surface and subsurface layers (0 m to 150 m), whereas MGII.B did not show a clear change at the different depths. This finding supports the suggestion that MGII.A and MGII.B might have different sensitivities to variations in temperature or nutrients (Hugoni et al., 2013). In addition to MGII.A and MGII.B, we also report a novel MGII lineage, MGII.C, according to our phylogenetic analysis. We showed that MGII.C archaea were mainly distributed in deep water but with relatively low abundance, which might explain why this lineage was rarely detected in previous studies. The distribution of MGII.C in other environments, such as sediment and fresh water, needs to be further confirmed.

**Table 5:** Comparison of archaeal assemblage composition in different geographic locations.

**Geographic location**	**Sampling time**	**Depth**	MGI^*^	MGII^*^	MGIII^*^	**References**
Arctic Ocean	07/2007	Surface	MGI:40%~56%	MGII.A:34~48%; MGII.B:2~3%	<0.4%	Galand et al., 2009
	01/2004 & 01/2007	Surface	MGI:48%~54%	MGII.A:20~36%; MGII.B:3~6%	<0.5%	
	07/2007 & 08/2007	410–1,000 m	MGI:28%~66%	MGII.A:0; MGII.B:10~20%	20~45%	
OMZ of the eastern tropical South Pacific	10/2005	Surface	MGI:0~2%	92~99%	0~4%	Belmar et al., 2011
	10/2005	100~350 m (Core of OMZ)	MGI: 1~50%	42~70%	0~33%	
South China Sea (SEATS)	10/2006	10 m	0	86%	9%	Tseng et al., 2015
	10/2006	100 m	22%	32%	32%	
	10/2006	1,000 m	13%	12.50%	45%	
	10/2006	3,000 m	14%	0	58%	
South China Sea (A5)	08/2009	Surface	MGI:7%	MGII.A:53%; MGII.B:27%	12%	Xia et al., 2015
	01/2010	Surface	MGI:32%	MGII.A:9%; MGII.B:57%	<2%	
South Pacific Gyre	11/2010 & 12/2010	Surface	0%	MGII.A:11~52%, MGII.B:47~88%	<1%	Yin et al., 2013
Northwest coastal Mediterranean Sea	08/2003 & 09/2003	Surface	4%	MGII.A:85%, MGII.B:12~20%	0%	Galand et al., 2010
	01/2003 & 01/2004	Surface	16~20%	MGII.A:14~20%, MGII.B:40~50%″	8~10%	
Northern Red Sea	02/2012	45 m	0%	97%	<3%	Hou et al., 2016
Western Antarctic Peninsula	02/2013 & 03/2013	Deep	50%	42%	8%	Signori et al., 2014

* *Percentage of the total archaeal community*.

Unlike MGII, MGI archaea were more abundant in the deep water of the Pacific Ocean. MGI archaea are known to be widely involved in ammonia oxidization and they are important for global carbon and nitrogen cycles. Our results indicate that MGI can be divided into four subgroups according to phylogenetic analysis. MGI.A and MGI.B, which were more abundant in subsurface and mesopelagic layers, were mainly contributed by OTU6 and OTU2, respectively. MGI.C, which had a higher abundance than the other three MGI subgroups, preferred deep water. The different geographic distributions of the various MGI archaea subgroups might suggest physiological differences among the MGI archaea strains.

Several previous studies have revealed that MGIII are more abundant in deep water (Table 5). This pattern was reiterated in most of our study stations. However, a relatively high abundance of MGIII was also detected in the surface waters of the SCS (20% of the sample reads) and the subtropical western Pacific Ocean (station SWP, 26% of the sample reads). Two previous studies also reported a relatively high abundance of MGIII (~9–10% of sample sequences) in the surface water of the SCS in the summer (Tseng et al., 2015; Xia et al., 2015). Together, these results suggest that MGIII might be a major component of the archaeal community in the surface water of some specific regions.

### High diversity of archaeal community in the oxygen minimum zone

One notable finding of this study was the high diversity of archaea that occurred in SEP2-500 m (i.e., in the core of the OMZ, DO concentration <0.8 μM; Cheung et al., 2016), where an extremely low oxygen concentration allowed the co-occurrence of both anaerobic and aerobic archaea. Thus, in addition to the typical marine archaea (MGI, MGII, and MGIII), several archaeal groups originally found in sediments or deep-hydrothermal vents, were also detected in this OMZ sample. These include MBGA, Bathyarchaea, and Parvarchaea (WCHD3-30, AR9-1, and YLA-114). Moreover, two methanogenic groups (I and II) and a methanotrophic archaeal group were found in the OMZ. In contrast, using the clone library method, Belmar et al. (2011) only detected MGI, MGII, and MGIII in the permanent OMZ of the eastern tropical South Pacific (ETSP) (Table 5). We suggest that the differences in archaeal community identified in the OMZ of the ETSP (Belmar et al., 2011) and the Costa Rica dome (this new study) might be due to the fact that the oxygen depleted layer of the two OMZs occur at different depths. MGIII were not abundant in the core of the ETSP OMZ (Belmar et al., 2011). However, we showed that MGIII could be a major archaeal lineage in low oxygen water. Most of the MGIII cells found in the OMZ were contributed by MGIII.B, suggesting that this sub-type might be able to cope with low oxygen concentrations more effectively than the other MGIII groups. In addition to MGIII, methanogenic archaea were found at high diversity and abundance in the OMZ off Costa Rica. This might suggest that similar to submarine sediments, hydrothermal seeps and cold seeps, the oxygen depleted water in OMZs might be an important source of methane. This finding supports a report from Levipan et al. (2007), which indicates that methylotrophic methane-producing archaea could account for up to 10% of the total prokaryotic rRNA in the OMZ. In addition, a recent study based on pyrosequencing of the *nifH* gene reported that the diazotroph community in the OMZ was mainly dominated by methanotroph-like diazotrophs (Cheung et al., 2016), which suggests a potential coupling between the nitrogen cycle and the methane assimilation pathway. We suggest that expansion of the OMZ due to global warming might result in an increase in the release of methane, which might aggravate global warming further.

Methanogenic archaea retrieved from the OMZ were from two different classes of Euryarcheota, i.e., Methanobacteriales and Methanomassiliicoccales. Methanogen-I were formed by sequences from Methanobacteriales and were more abundant and diverse. This archaeal group is usually found in marine sediment and deep-sea hydrothermal fields. Methanogen-II were represented by strain Kjm51a, which is normally enriched in anaerobic sludge and is a methanol-reducing hydrogenotrophic methanogen (Iino et al., 2013). To our knowledge, our study is the first report of this archaea in seawaters, although it has been found in the hydrothermal sediments of the Guaymas Basin in the Gulf of California (McKay et al., 2016; NCBI number: KP091025). As different methanogenic archaea co-occurred in the core of the OMZ off Costa Rica, this suggests that the deep OMZ might be a hotspot of methanogenic organisms.

The methanotroph-ANME-2 detected in this study is closely related to the sequence found in the Black Sea water column (Vetriani et al., 2003). In addition, whereas ANME-1 (another lineage of methanotrophic archaea) have previously been found to be more important in deep water (Carlström et al., 2016), no ANME-1 related sequence was detected in our study, indicating that this might have a narrower niche than ANME-2.

It is interesting that Marine Benthic Group A (MBGA) and Bathyarchaeota (formerly MCG), which are typical archaea of marine sediments (Kubo et al., 2012; Oni, 2015), were abundant in SEP-500 m. This is consistent with a previous study, which reported that the archaeal community in the OMZ includes archaeal groups from diverse seafloor environments (Ulloa et al., 2013). It has been reported that Bathyarchaea are one of the most active archaeal groups in the deep marine biosphere and globally important in sedimentary processes (Kubo et al., 2012). This archaea group has been shown to be abundant in the subsurface of marine sediments (Biddle et al., 2006), and it is suggested to play a role in the fermentation of refractory carbon (Kubo et al., 2012), such as aromatic compounds (Meng et al., 2014). Although Bathyarchaea contain at least 21 subgroups (Kubo et al., 2012; Fillol et al., 2016), in our study they only occurred in the OMZ sample and displayed a low diversity (i.e., just 3 OTUs), suggesting that the Bathyarchaea subgroups might have different physiological requirements. Whether MBGA and Bathyarchaea are also abundant in other OMZs needs further investigation. Parvarchaea are ultra-small size (<500 nm diameter) organisms, which were first found in a Californian mine (Baker et al., 2010), but have since been reported to occur in marine sediment (Costa et al., 2015). In addition, more recently it has been suggested that Parvarchaea might be true perchlorate respiring organisms (Carlström et al., 2016). To our knowledge, we are first to report Parvarchaeota in seawater. However, how and why all of these benthic archaea (MBGA, Bathyarchaeota, and Parvarchaeota) can thrive in the core of the OMZ, and how they function in biogeochemical cycles requires further study.

In summary, our results demonstrate remarkable differences in archaeal community composition between the surface and deep layers of the oceanic waters regardless of their geographical location. The archaeal communities in the surface water showed much higher geographic variation than those in the deep waters. This suggests that the dramatic differences in the physical and chemical conditions in the surface waters of the various geographic locations (e.g., subtropical oligotrophic warm water vs. subarctic high productive water) are the main forces that determine the archaeal composition, rather than the geographic distance. Our results also showed that surface archaeal communities in the high latitude cold water displayed a lower diversity than those in the subtropical warm waters, and that subgroups of MGI and MGII had different depth preferences. Further studies using the metagenomic sequencing method might reveal the physiological characteristics of the different archaeal groups and the reasons for the niche differentiation of the MG subgroups. Finally, our results highlight the importance of the OMZ as a hotspot of archaeal biodiversity. The low oxygen environment of the OMZ allows the co-occurrence of aerobic and anaerobic archaea, and the detection of both methanogenic and methanotrophic archaea suggests that this is an important site for methane cycling in the ocean.

## Author contributions

HL designed the study, and XX and WG performed the experiments. Data were analyzed by XX in collaboration with WG and HL. XX and HL wrote the manuscript, including comments from WG. All the authors reviewed and approved the final version of the manuscript.

### Conflict of interest statement

The authors declare that the research was conducted in the absence of any commercial or financial relationships that could be construed as a potential conflict of interest.
